# Extensive scalp soft-tissue reconstruction with free flaps: A simplified therapeutic algorithm for donor site selection based on a retrospective analysis

**DOI:** 10.1016/j.jpra.2024.11.003

**Published:** 2024-11-17

**Authors:** Raúl Martínez Peral, Josep Oriol Roca Mas, Gonzalo Joaquín Soroa Moreno, Adela Álvarez Río, Antonio Ansó Jiménez, Daniel Navarro Sánchez, Ivan Monge Castresana, Jaime Estrada Cuxart

**Affiliations:** Department of Plastic & Reconstructive Surgery, Hospital Universitario Son Espases, Palma de Mallorca, Balearic Islands, Spain

**Keywords:** Scalp reconstruction, Free flap, Omental flap, Anterolateral thigh flap, Latissimus dorsi flap, Radial forearm flap

## Abstract

**Introduction:**

Extensive scalp defects present a significant reconstructive challenge due to the complex needs of patients that are often beyond the scope of conventional therapies, which makes free flaps the most reliable solution. Despite the variety of free flaps available for such cases, there is a lack of clear criteria for selecting the most suitable option. The primary objective of this study was to provide a simplified guide for the selection of donor sites for free flaps for achieving optimal reconstruction outcomes.

**Materials and Methods:**

A retrospective study was conducted on 15 patients who underwent scalp reconstruction with free flaps between 2017 and 2022: 4 latissimus dorsi (LD), 4 omental (OM), 5 anterolateral thigh (ALT), and 2 radial forearm free (RFF) flaps. Reconstructive and postoperative data for all patients were collected, evaluated, and compared.

**Results:**

The mean defect size to be restored was 110.60 ± 14.55 cm² (LD 162.23 ± 23.1 cm^2^, OM 141.68 ± 11.80 cm^2^, ALT 73.83 ± 14.69 cm^2^, and RFF 37.13 ± 4.88 cm^2^). Seven complications were reported, with partial flap loss being the most common: LD *n* = 2 and OM = 3. Mean healing time of the donor and recipient sites was 2.53 ± 0.27 and 1.8 ± 0.31 months, respectively, with OM having the longest average period for recipient site healing (3.65 ± 0.24 months).

**Conclusion:**

Reconstructing extensive scalp defects requires careful consideration of critical factors such as defect size, donor tissue availability, need for adjuvant therapies, and patient comorbidities when selecting a flap. This underscores the importance of tailored approaches to enhance clinical outcomes. We propose a simplified algorithm for free flap selection to streamline the decision-making process in complex cases.

## Introduction

Extensive scalp defects can have multiple etiologies, with the most frequently reported ones arising from oncological resections, especially cutaneous tumors, followed by trauma, burns, inflammation, infections, radiation damage, and other causes.^1^^,^^2^ The scalp region represents a complex anatomical area composed of several tissue layers (skin, subcutaneous tissue, galea aponeurotica, loose areolar tissue, and periosteum) of variable thickness irrigated by the branches of the external and internal carotid arteries.^3^ These layers may be compromised to a greater or lesser degree, affecting the bone tissue, and underlying meningeal structures. As the number of affected structures increases, the difficulty in achieving successful reconstruction of the area significantly rises.

The primary objective in addressing the reconstruction of extensive scalp defects is to provide stable and well-vascularized tissue coverage that ensures the protection of the cranial vault, meninges, and brain tissue, while also aiming for an aesthetic appearance with minimal impact on the patients' quality of life. Selecting the optimal reconstruction strategy demands a thorough evaluation of the defect's location, size, and depth, as well as the patient's comorbidities and potential need for adjunct treatments such as radiotherapy. These factors can significantly influence the success of the chosen coverage by increasing the risk of local complications and impacting the long-term viability of the reconstruction.

Therapeutic armamentarium for scalp defect reconstruction includes a wide range of techniques. Primary closure or secondary intention healing may be sufficient for small defects (<5 cm^2^). For medium-sized defects (<30 cm²), local flaps (such as advancement, rotation, and transposition) can be used to rearrange viable adjacent tissues to cover the defect. There have been cases describing the restoration of defects of up to 100 cm² using large transposition flaps, albeit with significant sequelae in the donor area.^4^ When the amount of tissue required exceeds that available locally, other options, such as partial-thickness skin grafts (STSG), acellular dermal matrices, or tissue expanders can be used.^5^, ^6^, ^7^ However, these reconstructive options face serious challenges in complex cases, as they require specific conditions, such as a well-vascularized recipient bed with intact periosteum, viability of adjacent tissues, limited extent of involvement, or the absence of complementary therapies that could compromise the recipient area.

In cases where the defect exceeds 100 cm², which surpasses the amount of tissue available for local redistribution, or if it lacks periosteum and involves significant bone exposure, including exposed dura mater and inadequate recipient bed, more complex reconstructive techniques are mandatory. In such cases, the use of free flaps is crucial. The most frequently used free flaps for the reconstruction of large scalp defects are the latissimus dorsi (LD) flap, anterolateral thigh (ALT) flap, omental (OM) flap, and radial forearm (RFF) flap.^8^, ^9^, ^10^, ^11^ However, decision-making regarding free flap donor site selection raises debate because of the absence of clear criteria indicating a preference for one flap over another. The primary objective of this retrospective analysis was to evaluate and compare the outcomes of the main free flaps used for scalp reconstruction at our center, with the aim of creating a simplified guide for tailoring each case and achieving optimal results.

## Materials and methods

We conducted a retrospective case series study of patients with scalp and cranial vault defects requiring reconstruction with free flaps between April 2017 and October 2023 at the Son Espases University Hospital (Mallorca, Spain). Informed consent was obtained from all the patients. Data collection included demographic and etiological information; superficial extent and depth of affected structures; reconstructive procedures (including flap type, recipient vessels, vessel grafts, and operating room [OR] time); adjuvant therapies; and complications for all patients (Table 1A, Table 1BA,B). Qualitative variables are shown as frequencies and percentages (Table 2), whereas quantitative variables are presented as the mean ± standard error of the mean (Table 3).

**Table 1A:** Baseline patient characteristics, operative, and postoperative details.

	Etiology	Extent (cm^2^)	Bone involvement	Free flap type	Recipient vessels	Vessel graft	STSG	OR time (min)
Case 1 70/male	Tumor (PDS)	161.7	Yes	Latissimus dorsi flap	Superficial temporal External jugular vein	Yes (vein)	Yes	360
Case 2 76/male	Tumor (SCC)	222.7	Yes	Latissimus dorsi flap	Occipital External jugular vein	Yes (vein)	Yes	412
Case 3 72/female	Tumor (SCC)	154	Yes	Latissimus dorsi flap	Facial Thyrolinguofacial trunk	Yes (vein)	Yes	400
Case 4 71/male	Tumor (SCC)	110.5	Yes	Latissimus dorsi flap	Superficial temporal External jugular vein	No	Yes	370
Case 5 71/female	Inflammatory	141.75	No	Omental flap	Facial Thyrolinguofacial trunk	Yes (vein + artery)	Yes	483
Case 6 73/male	Tumor (SCC)	117	No	Omental flap	Superficial temporal External jugular vein	Yes (vein)	Yes	475
Case 7 55/male	Tumor (SCC)	173.45	No	Omental flap	Superficial temporal External jugular vein	No	Yes	370
Case 8 68/male	Tumor (SCC)	134.5	No	Omental flap	Superficial temporal External jugular vein	Yes (vein)	Yes	390
Case 9 64/female	Trauma	28.26	Yes	Anterolateral thigh flap	Superficial temporal External jugular vein	Yes (vein + artery)	No	400
Case 10 45/male	Tumor (SFT)	65.4	No	Anterolateral thigh flap	Superficial temporal External jugular vein	No	No	412
Case 11 35/male	Tumor (SCC)	71.5	Yes	Anterolateral thigh flap	Superficial temporal External jugular vein	No	No	425
Case 12 72/male	Tumor (SFT)	119	Yes	Anterolateral thigh flap	Superficial temporal External jugular vein	Yes (vein)	No	278
Case 13 74/male	Tumor (SCC)	85	Yes	Anterolateral thigh flap	Superficial temporal External jugular vein	Yes (vein)	No	325
Case 14 71/female	Inflammatory	42	Yes	Radial forearm flap	Superficial temporal External jugular vein	No	No	228
Case 15 76/male	Tumor (SCC)	32.25	Yes	Radial forearm flap	Superficial temporal Cephalic vein	No	No	342

PDS, pleomorphic dermal sarcoma; SCC, squamous cell carcinoma; SFT, solitary fibrous tumor; STSG, split-thickness skin grafts.

**Table 1B:** Baseline patient characteristics, operative, and postoperative details (continued).

	Complications	Reintervention	Adjuvant therapy	Hospital stay (d)	Follow-up time (mo)	Recipient site healing time (mo)	Donor site healing time (mo)
Case 1 LD	Hematoma (conservative)	No	Radiotherapy (60 Gy)	11	20	1	1.5
Case 2 LD	Partial flap loss (dressings)	No	No	44	16	2	3
Case 3 LD	No	No	Radiotherapy (60 Gy)	8	20	1	3
Case 4 LD	No	No	No	10	22	2	2
Case 5 OM	Partial flap loss (dressings)	No	No	9	18	4	3
Case 6 OM	No	No	No	11	22	3	2
Case 7 OM	Partial flap loss (dressings)	No	No	11	20	3.5	2
Case 8 OM	Partial flap loss (STSG)	Yes (1)	No	11	18	4	2
Case 9 ALT	No	No	No	25	18	1	3
Case 10 ALT	No	No	Radiotherapy (60 Gy)	21	16	1	2.5
Caso 11 ALT	No	No	Radiotherapy (60 Gy)	13	20	1	2
Case 12 ALT	No	No	No	17	18	1	2
Case 13 ALT	Infection (meningitis)	Yes (1)	No	35	18	1.5	2
Case 14 RFF	No	No	No	11	14	1	6
Case 15 RFF	No	No	Radiotherapy (60 Gy)	17	16	1	2

ALT, anterolateral thigh; LD, latissimus dorsi; OM, omental; RFF, radial forearm.

**Table 2:** Qualitative variables expressed in frequencies and percentages grouped by free flap.

		Latissimus dorsi flap (*n* = 4)	Omental flap (*n* = 4)	Anterolateral thigh flap (*n* = 5)	Radial forearm flap (*n* = 2)
Bone involvement	No	2	3	1	0
	Yes	2	1	4	2
Recipient artery	Superficial temporal	2	3	5	2
	Facial	1	1	0	0
	Occipital	1	0	0	0
Recipient vein	External jugular vein	3	3	5	1
	Thyrolinguofacial trunk	1	1	0	0
	Cephalic vein	0	0	0	1
Vein graft	No	0	1	2	2
	Yes	4	3	3	0
Complications	No	2	1	5	2
	Yes	3	3	1	0
	Partial flap loss	2	3	0	0
	Total flap loss	0	0	0	0
	Infection	0	0	1	0
	Hematoma	1	0	0	0
	Reintervention	0	1	1	0
Radiotherapy	No	2	4	3	1
	Yes	2	0	2	1

**Table 3:** Quantitative variables expressed as mean ± standard error of mean, grouped by free flap.

	LD	OM	ALT	RFF	Mean
Extent (cm^2^)	162.23 ± 23.1	141.68 ± 11.80	73.83 ± 14.69	37.13 ± 4.88	110.60 ± 14.55
Operating room time (min)	390.5 ± 12.52	429.5 ± 30.39	368 ± 28.42	285 ± 57	379.33 ± 17.54
Hospital stay (d)	18.25 ± 8.61	10.5 ± 0.5	22.2 ± 3.77	14 ± 3	16.93 ± 2.70
Follow-up (mo)	19.5 ± 1.26	19.5 ± 0.96	18 ± 0.63	15 ± 1	18.4 ± 0.59
Recipient site healing time (mo)	1.5 ± 0.29	3.63 ± 0.24	1.1 ± 0.1	1	1.87 ± 0.30
Donor site healing time (mo)	2.13 ± 0.375	2.25 ± 0.25	2.3 ± 0.2	4 ± 2	2.53 ± 0.27

## Results

Fifteen patients with extensive scalp defects who underwent reconstruction using free flaps were included. The mean age was 66.2 ± 3.09 years, comprising 12 men (80.0 %) and 3 women (20.0 %), with a mean follow-up time of 18.4 ± 0.59 months. The primary etiology of the defects was skin tumor resection (*n* = 12, 80 %), mainly squamous cell carcinoma (*n* = 8, 53.33 %), solitary fibrous tumor (*n* = 2, 13.33 %), and pleomorphic dermal sarcoma (*n* = 2, 13.33 %). Other etiologies included inflammatory (*n* = 2, 13.33 %) and traumatic (*n* = 1, 6.67 %) causes. Fifteen free flaps were used for the reconstruction: 4 LD, 4 OM, 5 ALT, and 2 RFF. The mean defect size was 110.60 ± 14.55 cm^2^ (LD 162.23 ± 23.1 cm^2^, OM 141.68 ± 11.80 cm^2^, ALT 73.83 ± 14.69 cm^2^, and RFF 37.13 ± 4.88 cm^2^), with 8 cases involving bone that required outer table milling and resection (LD *n* = 2, OM *n* = 1, ALT *n* = 4, and RFF *n* = 2).

The mean surgical time was 378 ± 17.34 min (LD 390.5 ± 12.52 min, OM 429.5 ± 30.39 min ALT 368 ± 28.42 min, and RFF 285 ± 57 min). Regarding arterial anastomoses, the superficial temporal vessels (*n* = 12), occipital artery (*n* = 1), and facial artery (*n* = 2) were employed as the recipient arteries. The external jugular vein (*n* = 12), thyrolinguofacial venous trunk (*n* = 2), and cephalic vein (*n* = 1) were predominantly used for venous anastomosis. Nine flaps required a saphenous vein graft to reach or adjust to the recipient vessels: 1 double-vein graft for the artery and vein (OM *n* = 1) and 8 single grafts for the vein (OM *n* = 2, LD *n* = 4, ALT *n* = 3). All flaps were harvested simultaneously, and no postural changes were necessary.

In the immediate postoperative period, 7 complications were recorded in 6 patients. These included 5 partial flap necroses, among which 4 were managed conservatively (LD *n* = 2 and OM *n* = 2) and 1 required debridement in the OR followed by coverage with local flap and split-thickness skin graft (OM *n* = 1). One infection in the recipient area was resolved with intraoperative debridement and thorough irrigation (ALT *n* = 1), while 1 hematoma of the donor site was conservatively managed (LD *n* = 1). No total flap loss or cerebrospinal fluid leakage was reported. The mean hospital stay was 16.93 ± 2.69 days (LD 18.25 ± 8.61 days, OM 10.5 ± 0.5 days, ALT 22.2 ± 3.77 days, and RFF 14 ± 3 days). Five patients (LD *n* = 2, ALT *n* = 2, and RFF *n* = 1) received adjuvant radiotherapy (each patient with a total dose of 60 Gy). The mean healing time of the recipient and donor area was 1.8 ± 0.31 months (LD 1.5 ± 0.29 months, OM 3.65 ± 0.24 months, ALT 1.1 ± 0.1 months, and RFF 1 months;) and 2.53 ± 0.27 months (LD 2.13 ± 0.375 months, OM 2.25 ± 0.25 months, ALT 2.3 ± 0.2 months, and RFF 4 ± 2 months), respectively.

## Discussion

**Figure 1:**
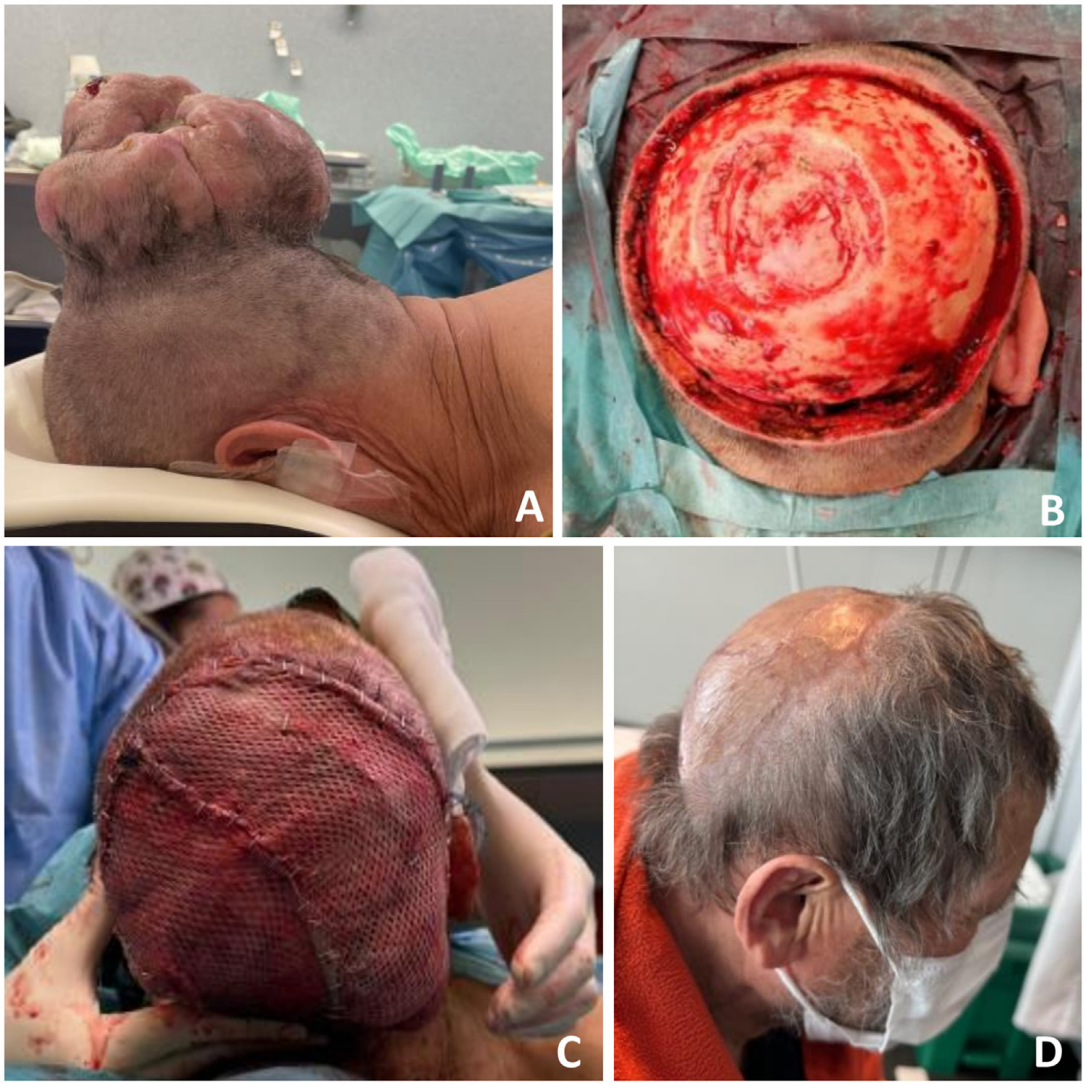
Case 2: latissimus dorsi flap. A, Scalp squamous cell carcinoma. B, Defect 222.7 cm^2^ after tumor resection. C, LD flap in-setting and grafted with split-thickness skin graft. D, Recipient site 4 months post-OP. LD, latissimus dorsi.

**Figure 2:**
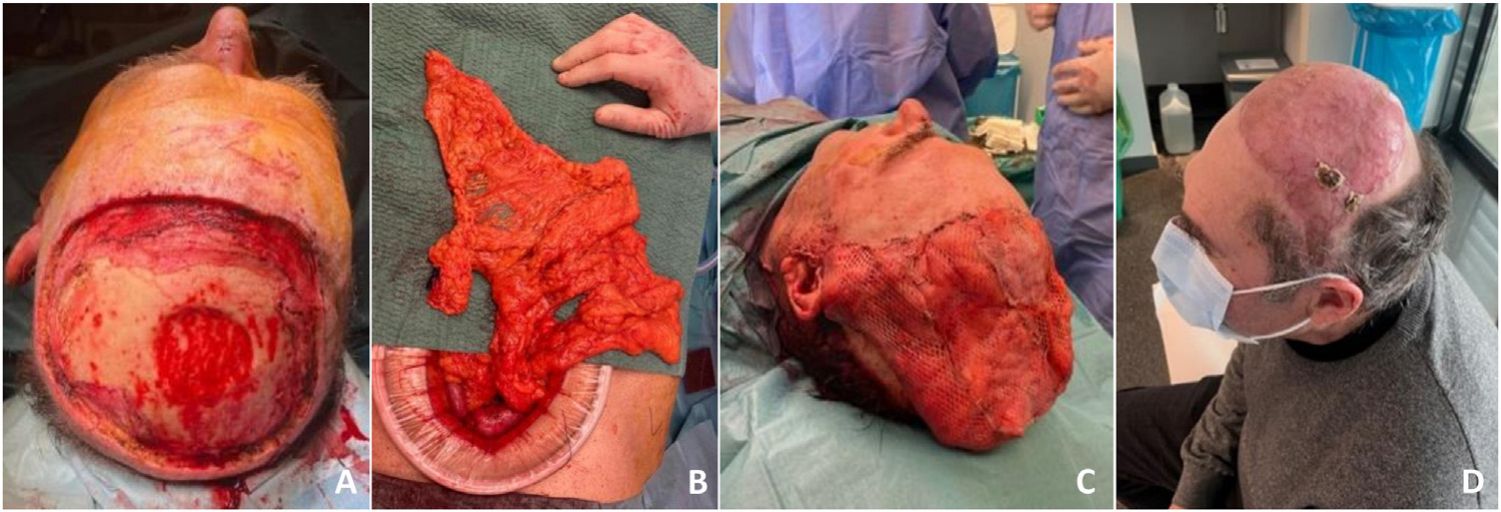
Case 3: omental free flap. A, Defect 117 cm^2^ after squamous cell carcinoma resection. B, Omental flap harvesting via midline laparotomy. C, Flap insetting and grafted split-thickness skin graft. D, Recipient site 3 months post-OP.

**Figure 3:**
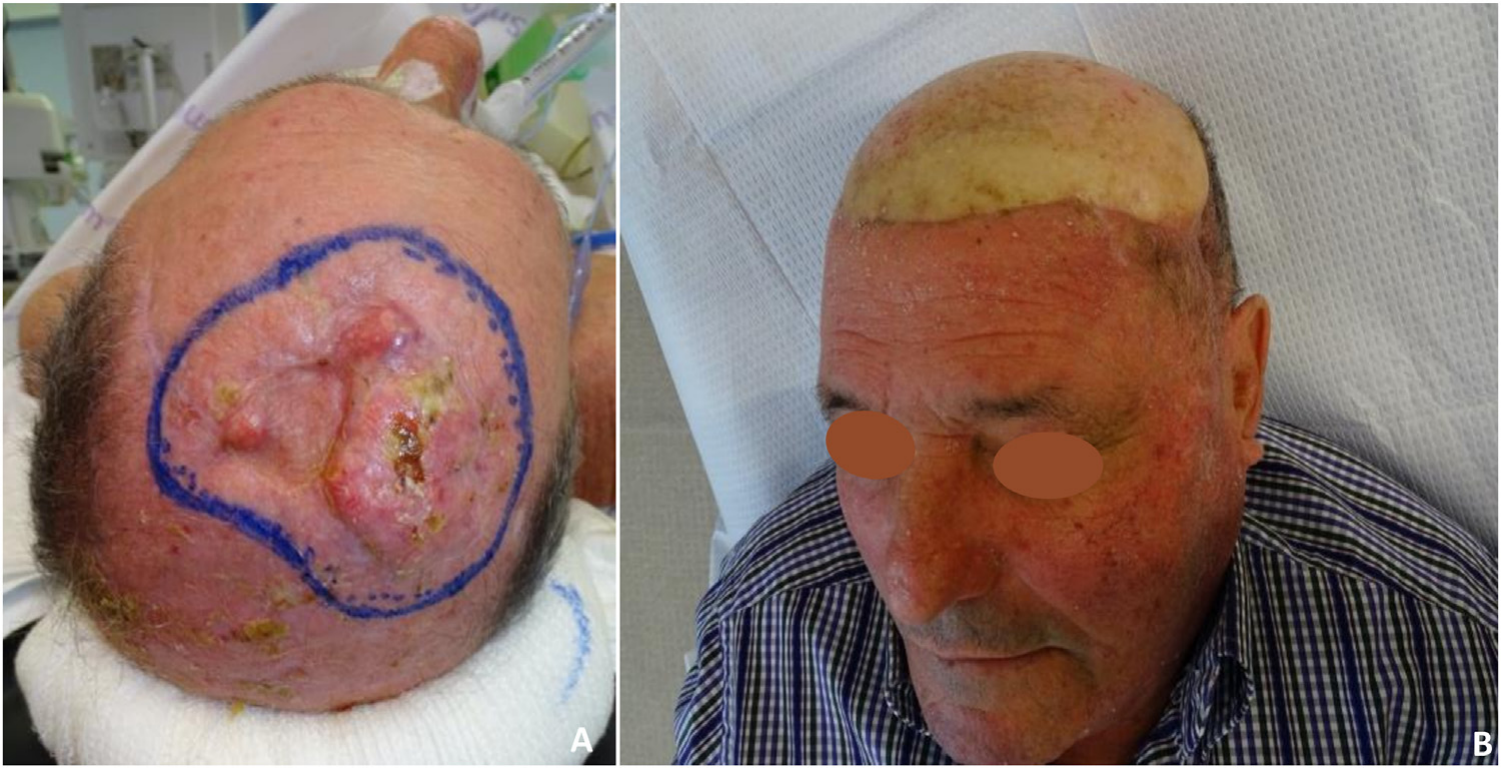
Case 10: anterolateral tight flap. A, Scalp fibrous solitary tumor (defect of 65 cm^2^ after tumor resection). B, Recipient site 4 months post-OP.

**Figure 4:**
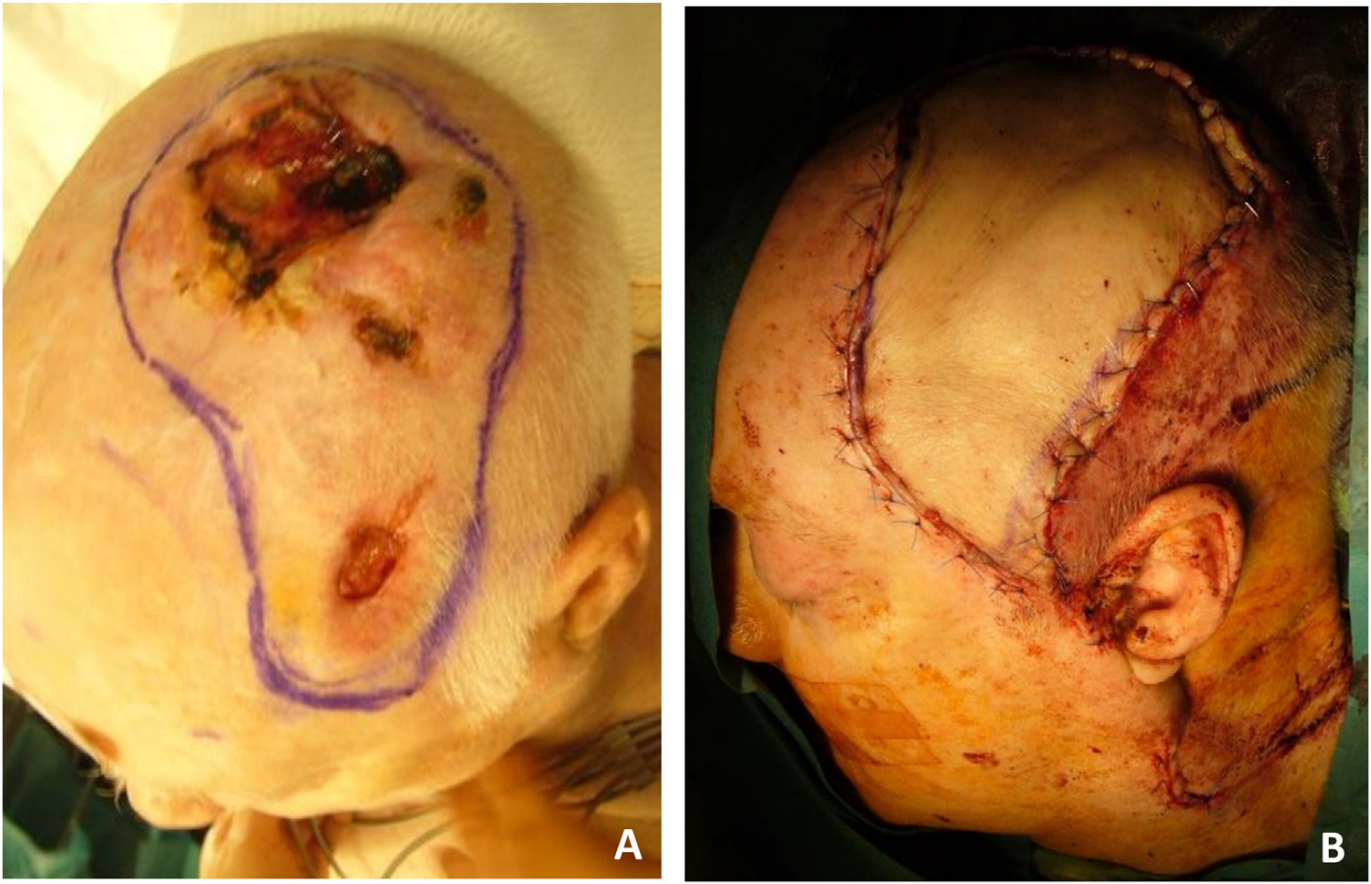
Case 14: radial forearm free flap. A, Scalp squamous cell carcinoma (42 cm^2^ defect size). B, Recipient site 6 months post-OP.

Reconstructing extensive scalp defects requires meticulous planning and consideration of multiple factors, including the size and location of the defect, number of anatomical structures involved, availability of donor tissue, quality of the surrounding skin, vascularization of the recipient area, need for adjuvant therapies, and patient comorbidities. These defects present a therapeutic challenge for the reconstructive surgeon because of the need to provide a significant amount of coverage to an area with limited adjacent tissue, and in several cases, because of the etiology of the injury, with a poorly vascularized recipient bed due to involvement of the periosteum, bone, and/or dura mater. Free flaps become the unique solution for covering these defects as they provide extensive, well-vascularized coverage independent of the nature of the recipient bed. Numerous free flaps have been reported for the microsurgical reconstruction of complex scalp defects, with the LD, ALT, OM, and RFF being the most commonly employed^8^, ^9^, ^10^, ^11^ (Figure 1, Figure 2, Figure 3, Figure 4). To ensure optimal scalp reconstruction, it is crucial to adapt the reconstructive approach to the individual characteristics of each patient, as there is no gold-standard free flap that predominates over the others.

### Extent

The LD and OM provide stable coverage for very large defects exceeding 100 cm², involving almost the entire scalp. In our series, the large defects had a mean of 162.23 ± 23.1 cm^2^ and 141.68 ± 11.80 cm^2^, respectively (Figs. 1,2). The ALT flap would be more suitable for the reconstruction of moderate-sized defects, between 50 and 100 cm² (73.83 ± 14.69 cm² in our study), provided that the donor site allows for direct closure, since the need to graft the donor site prolongs bed rest along with the subsequent negative consequences, especially in older patients (Figure 3). Finally, the RFF would be a more suitable option for smaller defects, between 25 and 50 cm² (37.13 ± 4.88 cm² in our study) (Figure 4). Larger defects typically require more extensive flaps, such as OM or LD flaps, combined with STSG. However, these larger flaps have a higher incidence of partial loss in the distal areas than smaller flaps, such as the ALT or RFF, due to the need for perfusion over a larger surface (*n* = 7/8, 62.5% vs. *n* = 1/7, 14.3 %). An effective strategy to address this issue is to perform intraoperative assessment of flap perfusion using indocyanine green fluorescence. This technique enables the real-time evaluation of vascular perfusion within the flap. If compromised perfusion is identified in any part, the specific area can be excised prior to the final inset, ensuring optimal outcomes.^12^ Nonetheless, it is important to bear this factor in mind during planning and preparation for the possible need for secondary procedures in the future, either to provide additional coverage with local flaps or to adopt a conservative management approach with prolonged follow-up in consultations.

### Recipient vessels

The choice of recipient vessels is critical for successful scalp reconstruction. The superficial temporal vessels are most frequently used due to their proximity (80 % of patients in our study).^13^^,^^14^ When these vessels are not viable, such as after radiation therapy or previous surgeries, alternatives such as the facial, superior thyroid, occipital, lingual, and transverse cervical arteries can be considered.^15^^,^^16^ These options often require additional techniques, including venous grafts, vein loops, or pedicle lengthening, due to their distance from the surgical site.^17^ In our study, alternatives including the facial artery (OM *n* = 1, LD *n* = 1) and occipital artery (LD *n* = 1) were used, with the latter being accessible in the lateral decubitus position. Ten cases required venous grafts from the greater saphenous vein: 8 for the external jugular vein (LD *n* = 3, OM *n* = 2, and ALT *n* = 3), 2 for the thyrolinguofacial trunk (LD *n* = 1 and OM *n* = 1), and 1 for the facial artery (OM *n* = 1). Although most flaps used for scalp reconstruction have long pedicle, extensive defects or mismatched vessel calibers often necessitate vascular grafts, particularly for the veins.^11^ Preoperative imaging with Doppler ultrasound or angioCT is vital for assessing vessel condition and ensuring readiness for vein graft harvesting if needed.^18^

### Need for adjuvant therapies

Another key element in scalp reconstruction planning is the etiology of the defect. Several cases are secondary to radical excision of tumors, predominantly cutaneous,^1^^,^^2^ some of which include adjuvant therapies such as radiotherapy in their treatment protocols to reduce locoregional recurrences and increase survival rates.^19^ In our series, 5 patients (LD *n* = 2, ALT *n* = 2, and RFF *n* = 1) received adjuvant therapy with radiotherapy (total dose protocol of 60 Gy). Radiotherapy induces secondary effects at the recipient site, causing vascular and tissue damage. When applied preoperatively, it promotes scar tissue formation and complicates the preparation and microvascular anastomosis of the recipient vessels. When applied postoperatively, it delays flap and recipient site healing, thereby, endangering flap viability.^20^ Although it is generally administered postoperatively to reduce the risk of complications, radiotherapy-induced vasculitis can cause delays in healing the recipient area.^21^^,^^22^ This factor must be considered because the healing time of the recipient site varies among the different types of flaps. Our study observed significant differences in the healing time between OM flaps compared to other flaps (3.63 ± 0.5 months vs. 1.1 ± 1.1 months). Therefore, we believe that omental flaps should be reserved for cases where adjuvant radiotherapy is not anticipated, whereas musculocutaneous or fasciocutaneous flaps should be used in patients for whom radiotherapy is indicated and a delay in healing is expected.

### Comorbidities

Microvascular scalp reconstruction is a complex process that requires the careful consideration of several factors, including the individual characteristics of the patient and potential complications associated with the use of different flap types. Although age should not be considered a contraindication by itself,^23^ it is important to consider other factors such as a history of previous surgeries, medication, and diseases that may compromise the viability of the flap. In the case of the OM, its harvest requires a midline laparotomy approach with the consequent formation of adhesions (no abdominal complications were recorded in our study), although it can also be harvested via laparoscopy.^24^^,^^25^ Therefore, patients with a history of intra-abdominal surgery present a relative contraindication for the use of this flap. However, in the case of the LD flaps, it is important to consider the reduction in functional capacity of the shoulder movements, which is particularly vulnerable for older patients with limited mobility or workers who require physical exertion. The same must be considered for the RFF in handworkers. An additional aspect to be considered is the possibility of developing local complications such as hematomas, seromas, and infections, as well as the need for graft coverage of the donor site, as seen with the ALT flap when direct closure is not achieved or the RFF. This also applies to the donor site in case of the LD or OM, which can lead to complications such as delayed healing, especially in older patients. In our series, only 1 complication at the donor site was reported: 1 hematoma which was managed conservatively (LD *n* = 1). The mean healing time for the donor site was 2.53 ± 0.27 months (LD 2.13 ± 0.375, OM 2.25 ± 0.25, ALT 2.3 ± 0.2, and RFF 4 ± 2 months).

### Simplified donor site selection algorithm

**Figure 5:**
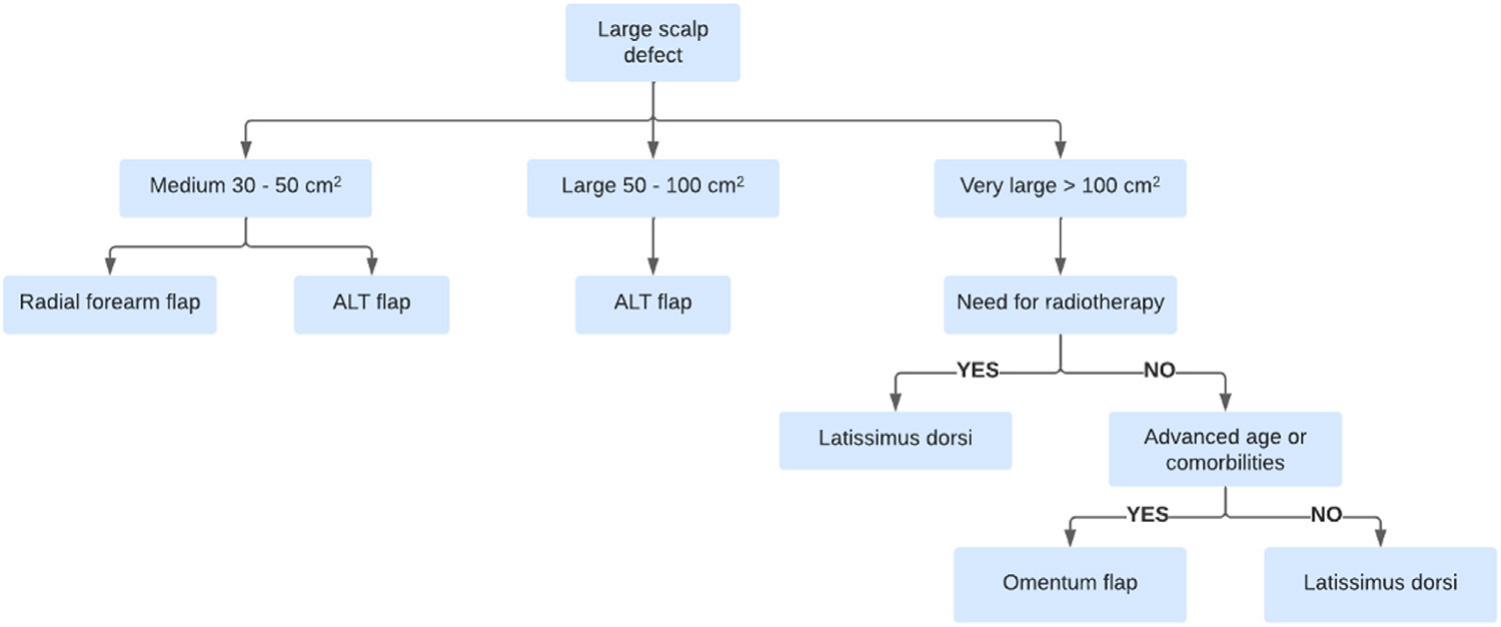
Therapeutic algorithm for free flap donor site selection.

Based on the aforementioned considerations, we propose a simplified algorithm (Figure 5) as a practical guide for selecting the donor site in the reconstruction of extensive scalp defects using free flaps. This algorithm considers critical factors such as defect size, need for adjuvant therapies, and patient comorbidities. Although the sample size may constrain the generalizability of our findings, the algorithm is designed to simplify and improve the efficiency of the reconstructive process, providing a structured approach that can support clinical decision-making even when individualized considerations are required.

## Conclusion

Reconstruction of extensive scalp defects is a complex challenge that requires thorough consideration of multiple factors, including the etiology of the defect, its size and depth, availability of donor tissue, and patient comorbidities. Free flaps, such as the OM, LD, ALT, and RFF flaps, are effective options for microsurgical scalp reconstruction. Each type of flap has its own advantages and considerations, emphasizing the importance of tailoring the reconstruction approach to the individual needs of each patient. This retrospective analysis highlights these considerations to provide guidance in selecting the donor site, with the aim of improving clinical outcomes and patient quality of life.
